# The role of digital tools and emerging devices in COVID-19 contact tracing during the first 18 months of the pandemic: a systematic review

**DOI:** 10.1093/eurpub/ckae039

**Published:** 2024-07-01

**Authors:** Brigid Unim, Irisa Zile-Velika, Zane Pavlovska, Luis Lapao, Mariana Peyroteo, Janis Misins, Maria João Forjaz, Paulo Nogueira, Tiziana Grisetti, Luigi Palmieri

**Affiliations:** Department of Cardiovascular, Endocrine-metabolic Diseases and Aging, Istituto Superiore di Sanità, Rome, Italy; Centre for Disease Prevention and Control of Latvia, Riga, Latvia; Centre for Disease Prevention and Control of Latvia, Riga, Latvia; UNIDEMI, Department of Mechanical and Industrial Engineering, NOVA School of Science and Technology, Universidade Nova de Lisboa, Caparica, Portugal; CHRC, Nova Medical School, Universidade Nova de Lisboa, Lisbon, Portugal; UNIDEMI, Department of Mechanical and Industrial Engineering, NOVA School of Science and Technology, Universidade Nova de Lisboa, Caparica, Portugal; CHRC, Nova Medical School, Universidade Nova de Lisboa, Lisbon, Portugal; Centre for Disease Prevention and Control of Latvia, Riga, Latvia; National Center of Epidemiology, Health Institute Carlos III and RICAPPS, Madrid, Spain; CHRC, National School of Public Health, Nova de Lisboa University, Lisbon, Portugal; Nursing Research, Innovation and Development Centre of Lisbon (CIDNUR), Nursing School of Lisbon, Lisbon, Portugal; Instituto de Saúde Ambiental (ISAMB), Laboratório para a Sustentabilidade do Uso da Terra e dos Serviços dos Ecossistemas—TERRA, Faculdade de Medicina, Universidade de Lisboa, Lisbon, Portugal; Department of Cardiovascular, Endocrine-metabolic Diseases and Aging, Istituto Superiore di Sanità, Rome, Italy; Department of Cardiovascular, Endocrine-metabolic Diseases and Aging, Istituto Superiore di Sanità, Rome, Italy

## Abstract

**Background:**

Contact tracing is a public health intervention implemented in synergy with other preventive measures to curb epidemics, like the coronavirus pandemic. The development and use of digital devices have increased worldwide to enhance the contact tracing process. The aim of the study was to evaluate the effectiveness and impact of tracking coronavirus disease 2019 (COVID-19) patients using digital solutions.

**Methods:**

Observational studies on digital contact tracing (DCT), published 2020–21, in English were identified through a systematic literature review performed on nine online databases. An *ad hoc* form was used for data extraction of relevant information. Quality assessment of the included studies was performed with validated tools. A qualitative synthesis of the findings is reported.

**Results:**

Over 8000 records were identified and 37 were included in the study: 24 modelling and 13 population-based studies. DCT improved the identification of close contacts of COVID-19 cases and reduced the effective reproduction number of COVID-19-related infections and deaths by over 60%. It impacted positively on societal and economic costs, in terms of lockdowns and use of resources, including staffing. Privacy and security issues were reported in 27 studies.

**Conclusions:**

DCT contributed to curbing the COVID-19 pandemic, especially with the high uptake rate of the devices and in combination with other public health measures, especially conventional contact tracing. The main barriers to the implementation of the devices are uptake rate, security and privacy issues. Public health digitalization and contact tracing are the keys to countries’ emergency preparedness for future health crises.

## Introduction

Contact tracing is the process of identifying and managing individuals who have been in the proximity of a person diagnosed with an infectious disease, in order to prevent additional transmission. It is ‘an essential public health measure to fight the coronavirus disease 2019 (COVID-19) pandemic, in conjunction with active case finding and testing and in synergy with other measures such as physical distancing’.^1^ Contact tracing has become a key element of strategies to control the spread of severe acute respiratory syndrome coronavirus-2 (SARS-CoV-2), and countries worldwide have increasingly looked to digital technologies in support of public health measures for contact tracing of COVID-19 cases.^1–4^ The use of emerging devices (e.g. smartphones), in combination with traditional methods of contact tracing, has offered new potential for health authorities to limit or interrupt chains of SARS-CoV-2 transmission.^1^ Such solutions are also featured as public advocacy measures and a means to adjust national public health and social measures.^5^

Digital contact tracing (DCT) typically uses smartphones or other devices (e.g. drones, eBracelets, thermal scans, artificial intelligence-based tools) and online platforms to monitor interactions between individuals and issue real-time alerts in case of contacts with COVID-19 cases. These devices are also deployed for patients’ remote management, research activities and the implementation of public health measures.^6^

The effectiveness of DCT depends on its integration into well-established testing and contact tracing infrastructures.^2^ Using digital tools in support of a comprehensive contact tracing strategy has shown promising results.^1^^,^^5^ However, there are still aspects that need to be addressed, such as the overall effectiveness of DCT solutions, including comparisons between manual and DCT, the impact of the community uptake, data privacy and security.^7^ Although the above questions have been studied from individual studies, a current systematic review that summarizes these findings is lacking.^8^ To this end, the effectiveness and impact of digital tools and emerging devices in COVID-19 contact tracing and their potential role in future health emergencies were assessed within the framework of the ‘Population Health Information Research Infrastructure’ (PHIRI)^9^ PHIRI was developed to facilitate and generate the best available evidence for research on the health and well-being of populations impacted by COVID-19.

## Methods

The study was performed according to the Preferred Reporting Items for Systematic Reviews and Meta-Analyses Statement.^10^ The search string (coronavir* OR corona virus* OR corona pandemic* OR betacoronavir* OR covid19 OR covid OR nCoV OR novel CoV OR CoV2 OR sarscov2 OR sars2 OR 2019nCoV OR wuhan virus*) AND (contact tracing OR contact tracing tool* OR contact tracing strategies OR mobile application* OR electronic device* OR population surveillance OR public health surveillance OR epidemiological monitoring OR infection control OR communicable disease control OR smartphone OR disease notification) was applied and adapted by two investigators on nine online databases (i.e. PubMed, Scopus, World Health Organization, Biomed Central, Web of Science, Cochrane Library, Chinese Center for Disease Control and Prevention, European Center for Disease Control and Prevention and Center for Disease Control and Prevention). Relevant articles were also identified from the reference list of excluded systematic reviews. The inclusion criteria were the following:

1. observational and modelling studies focusing on DCT of COVID-19 in the population, published in English from January 2020 to October 2021 and providing quantitative data. The initial 18 months of the pandemic were considered as most countries discontinued the use of contact tracing devices thereafter, due to low adoption rates.^6^ Also, contact tracing among the general population ceased to be an effective strategy against the Omicron variant due to its high transmission rates; priority was given to high-risk settings (e.g. hospitals) and contacts (e.g. vulnerable populations)
2. mobile devices or web platforms used for DCT
3. population-based contact tracing, including nursing homes and long-term care facilities
4. modelling studies using real-world data or hypothetical populations

Data extraction from the included studies was performed using an *ad hoc* extraction form, distinguishing population-based studies (real-world contact tracing) from modelling studies. The main sections of the form for population-based studies included the following:

1. general characteristics of the study (first author and year of publication, study setting, design and period, name and type of the contact tracing device/digital platform, technology employed, definition of contact and comparisons with other digital tools)
2. uptake rate by the population (percentage of persons who downloaded and actively used the app, and of all positive tests that occurred among app users)
3. security, ethical and privacy considerations (e.g. privacy from authorities and contacts, user consent; equity, harms from false positive/negative results; cyber attack protection through passwords, anonymization techniques, centralized or decentralized system).

Besides these sections, the extraction form for modelling studies included information about the type of model, study population, type of intervention (e.g. DCT alone or in combination with other measures, manual tracing, isolation, social distancing), and comparisons with other interventions (e.g. no intervention, lockdown, social distancing without contact tracing). Other aspects considered were sensitivity analysis, communication technologies used by the devices, data sources, privacy and security issues of the tools, definition of contacts, COVID-19 test specificity and sensitivity and public availability of the model code.

Besides the uptake rate in population-based studies, the effectiveness of DCT was evaluated for both study types using metrics adapted from the Indicator Framework^5^:

1. the number of close contacts of COVID‐19 cases and of close contacts per index case identified
2. the number of laboratory-confirmed COVID-19 cases detected from close contacts
3. reduction of the effective reproduction number or reduction of COVID-19 infections.

The quality assessment of the studies was performed with the Effective Public Health Practice Project tool for population-based studies^11^ and the Consolidated Health Economic Evaluation Reporting Standards (CHEERS) checklist^12^ for modelling studies. Questions not relevant to non-economic modelling studies were omitted from the CHEERS checklist (i.e. 1, 6, 8–14 and 19–21). The results of the current study are presented as a qualitative synthesis, due to the heterogeneity of the included records.

## Results

**Figure 1:**
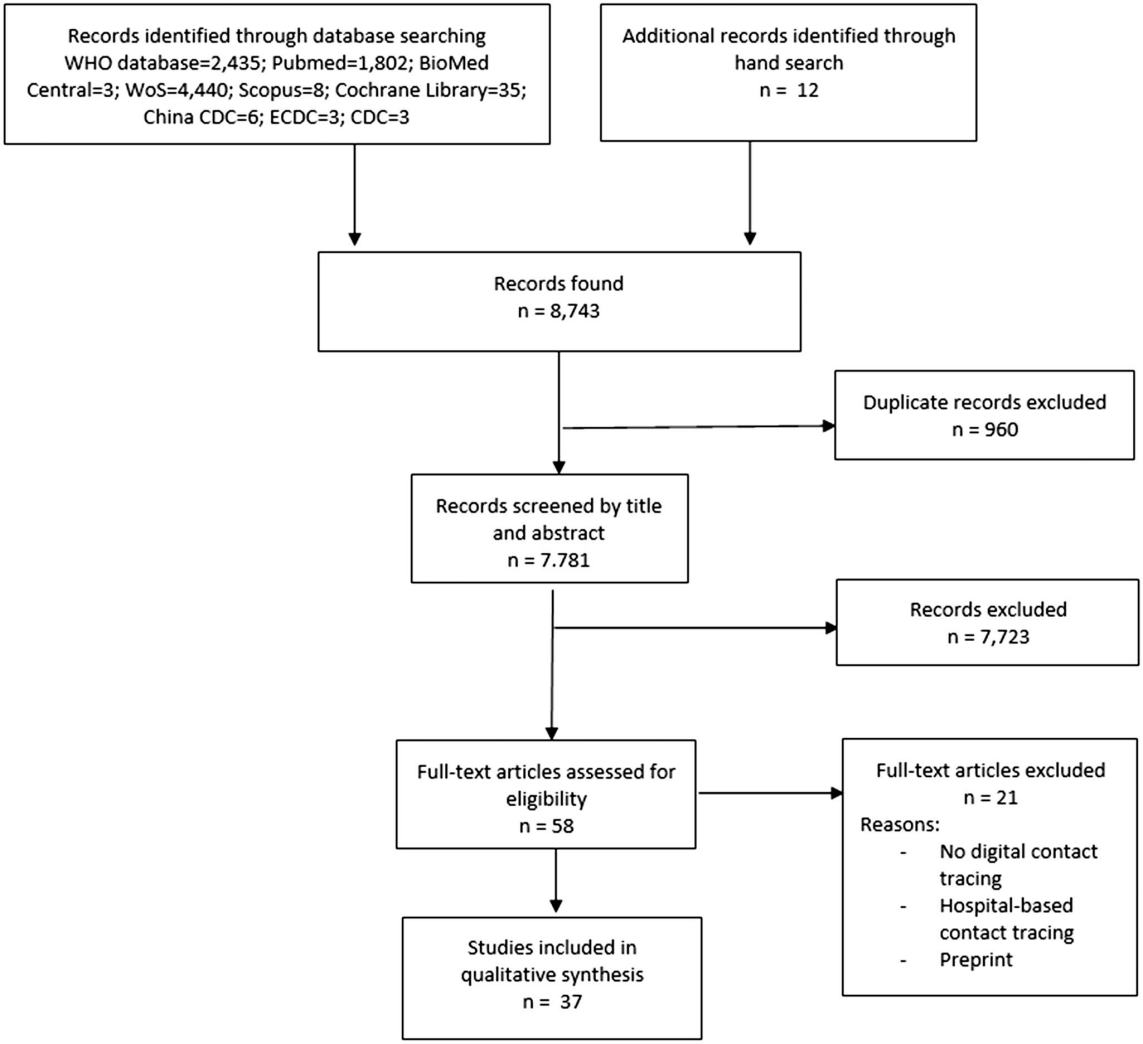
Flow diagram depicting the study selection procedure on digital contact tracing during the first 18 months of the COVID-19 pandemic

The search strategy identified 8743 records (figure 1), of which over 7000 were screened by title and abstract after duplicate removal. Fifty-eight full-text articles were assessed for eligibility with the elimination of 21 records, leaving 37 articles (13 population-based and 24 modelling studies) in the systematic review.

### Population-based studies

Most population-based studies (10/13) were published in 2020 and in Asian countries (6/13) (table 1); one multinational study was conducted in Asia, Europe and the USA.^13^ The majority (9/13) had a cross-sectional design, of which two studies had also a cohort design.

**Table 1:** General characteristics of the population-based studies included in the systematic review on COVID-19 digital contact tracing

References	Study setting	Study design	**Name of the contact tracing device/electronic platform**	Type of device/platform	Technology employed	Close contacts of COVID‐19 cases; close contacts per index case	Laboratory-confirmed COVID-19 cases detected from close contacts *N* (%)	**Reduction of effective reproduction number (95%CI)** ^a^ **/COVID-19 infections**	Privacy issues	Ethical issues	Security measures
Bae^17^	Korea	Cross-sectional	nr^b^	Smartphone	Manual contact tracing + GPS^c^ of mobile phones + credit card transactions + CCTV^d^	1687 (14.5 ± 26.3 close contacts per index case)	108 (6.4%)	Rt = 6.1 at the beginning of the outbreak; Rt < 1, 2 days after the epidemiological investigation was launched. Rt current outbreak: 0.79 (95%CI 0.66–0.93)	Individual consent was not applicable	The data were collected as part of an epidemiological investigation of KCDC,^e^ and ethical approval was not applicable. The use of the data was approved by KCDC.^e^	nr^b^
Barrett^f^	Ireland	Cross-sectional	Automated text messaging system	Mobile telephone + text broadcasting software	Text message-based system	1336	35 (2.6%)	nr^b^	Verbal consent provided by all participants; option to withdraw from active surveillance at any time; compliance with the GDPR^g^	nr^b^	nr^b^
Chen^16^	Taiwan	Cross-sectional	Multiple systems	Smartphone + CCTV^d^+ credit card terminals + geotracking system	Manual contact tracing + GPS^c^ + credit card transactions + CCTV^d^ + GPS^c^	627,386	None of the symptomatic or hospitalized contacts were confirmed as cases	nr^b^	The mobile position method does not infringe on individual confidentiality	Under the Taiwan Infectious Disease Control Act (2007), authorization or consent to the retrieval of individual information can be waived	nr^b^
Fetzer^f^	UK (England)	Ecological, natural experiment	NHS^h^ COVID-19 app	Smartphone	Google Apple EN^k^ system + Android and iOS^i^operating systems + Bluetooth	nr^b^	nr^b^	63% reduction in new infections; 66% reduction in COVID-19-related deaths	nr^b^	nr^b^	Centralized system
Jian^4^	Taiwan	Cross-sectional	TRACE (national contact tracing platform)	Smartphone-based system + web app	Manual contact tracing + GPS^c^ + web-app contact management system	8051 close contacts (16.5 close contact/index case; 95%CI 13.9–19.1)	147 (1.8%)	nr^b^	Voluntary basis; the database containing personal information will be deleted in 6 months and cannot be used for other purposes	nr^b^	Centralized system
Krueger^3^	USA	Cross-sectional	Sara Alert (symptom monitoring system)	Web-based symptom monitoring tool	Manual contact tracing + automated monitoring via web-based symptom monitoring tool	1622 close contacts (2.9 per index case; 95%CI 0–31)	127 (7.8%)	nr^b^	Voluntary basis: 96.4% monitored contacts chose automated over direct symptom monitoring	nr^b^	nr^b^
Kwon^f^	Korea	Experimental study (cohort)	Epidemic Investigation Support System	Smartphone + CCTV^d^ + credit card terminals	GPS^c^ + credit card transactions + CCTV^d^	13	2 (15.4%; 95%CI 8.3–22.5)	na^b^	nr^b^	nr^b^	nr^b^
Mack^14^	USA	Cross-sectional	KINEXON	Wearable proximity device	Manual contact tracing + proximity device	189	20 (11%)	COVID-19 transmission is reduced through environmental change, increased personal protection, avoidance of high-risk interactions	nr^b^	nr^b^	nr^b^
Salathé^2^	Switzerland	Cross-sectional, cohort	SwissCovid app	Smartphone + FOPH^j^ computer server	EN^k^ framework via Bluetooth	185 close contacts in the cohort study (0.24 per index case; 95%CI 0.20–0.27)	nr^b^	nr^b^	Notifications are shown only on the phone and are not forwarded to a server; voluntary basis	nr^b^	Decentralized system
Urbaczewski^13^	China, Germany, Italy, Singapore, South Korea, USA	Ecological	nr^b^	Smartphone	GPS,^c^ Bluetooth	nr^b^	nr^b^	nr^b^	Not mandated apps: China, Germany, Italy, and the USA; mandate apps: South Korea, Singapore	nr^b^	Centralized systems: Italy, Singapore, the USA, China; Centralized + decentralized systems: the USA; unknown: South Korea
Wymant^33^	UK (England, Wales)	Ecological	NHS^h^ Covid-19 app	Smartphone	Google Apple EN^k^ system + Android and iOS^i^ operating systems + Bluetooth	1.7 million (4.2 per index case)	6% (95%CI 5.96–6.09%)	On average, each confirmed COVID-19-positive individual who consented to notification of their contacts through the app prevented one new case	Privacy-preserving Google Apple EN system; user’s approval required for digital contact tracing in case of positive COVID-19 test results	nr^b^	nr^b^
Yamamoto^15^	Japan	Cross-sectional, cohort	K-note (PHR^l^-based health observation app)	Smartphone or tablet app integrated with PHR^l^-based app	Manual contact tracing + automated monitoring via digital symptom monitoring tool + email + manual data visualization (Excel macro)	Ccohort: 72; cross-sectional: nr^b^	nr^b^	na^m^	Voluntary basis; protection of personal information; users’ consent for data sharing	nr^b^	Decentralized system
Zhang^18^	China	Cross-sectional	na^b^	GPS^c^ + different electronic systems	Manual contact tracing + mobile phone location data + big data technology + electronic payment history	Five out of 100 secondary cases (5%)	nr^b^	nr^b^	nr^b^	nr^b^	nr^b^

a CI, confidence interval.

b nr, not reported.

c GPS, Global Positioning System.

d CCTV, closed-circuit television.

e KCDC, Korean Centers for Disease Control and Prevention.

f Supplementary material S1.

g GDPR, General Data Protection Regulation.

h NHS, National Health Service.

i iOS, iPhone Operating System.

j FOPH, the Swiss Federal Office of Public Health.

k EN, exposure notification.

l PHR, personal health record.

m na, not applicable.

The study population (data not presented in table 1) was the general population in all studies, except in one^14^ with National Football League players and staff members as the main targets. The uptake rate of the devices was evaluated through the number of downloads (range 27.44–100%), active users (61.5–100%) and positive tests among app users (0–14.8%). All studies, except one,^13^ provided a definition of contact that included physical distancing, duration and frequency of the encounters.

Smartphones, frequently in combination with other tracking systems, were the main tools evaluated in the studies (10/13), while wearable devices^14^ and web-based monitoring tools^3^ were examined in one study each. Data exchange between devices was based on Global Positioning System (GPS) and Bluetooth technologies. Geolocalization was performed through a combination of mobile phone operating systems (Android and Apple), closed-circuit television (CCTV), text messaging and electronic payment systems. Manual contact tracing was also evaluated (7/13). Comparisons were made, except in 9/13 studies, with other digital tools, traditional contact tracing or no contact tracing. Comparisons between interventions revealed the effectiveness of combining digital and traditional contact tracing. DCT has a similar capacity at identifying contacts of index cases as classic contact tracing, provided that the index case and the exposed contacts use the app.^2–4^^,^^15^ Also, the workload due to monitoring of infected close contacts decreases.^4^^,^^15^^,^^16^ The effectiveness of conventional epidemiological investigations in combination with DTC, isolation or testing was also observed.^14^^,^^17^^,^^18^

Regarding the effectiveness of contact tracing, the number of identified close contacts ranged across the studies from 5 to 1.7 million, and from 0.24 to 16.5 close contacts per index case. The laboratory-confirmed COVID-19 cases detected from close contacts ranged from zero to 15.4%. The effective reproduction number was reported in one study: it was above six at the beginning of the outbreak and lower than one after the launch of the epidemiological investigation. Three studies indicated a substantial decrease in COVID-19 infections (table 1).

Information about privacy, ethical issues and security measures was available in nine, two, and five studies, respectively. The use of monitoring devices was mandatory in South Korea, Singapore and Taiwan. In Taiwan, after the outbreak of SARS in 2007, authorization or consent for the retrieval of personal information related to the outbreak of emerging infectious diseases, such as SARS-COV-2, could be waived. In South Korea, COVID-19-related data were collected as part of the epidemiological investigation of the Korean Centers for Disease Control and Prevention, and individual consent was not applicable. Centralized server systems for the storage and processing of the collected data were deployed in Italy, Singapore, the USA, China, Taiwan and the UK. Decentralized systems, less subjected to data breaches, were implemented in Switzerland, Japan and some States in the USA (table 1).

### Modelling studies

The modelling studies (table 2) were conducted mostly in 2020 (16/24) and in Europe (8/24); the setting was not specified in seven studies. Various models were deployed, including compartmental models such as Susceptible-Infected-Removed (SIR) and Susceptible-Exposed-Infectious-Removed (SEIR) and their adapted versions, agent-based models and individual-based models. The general population was the main target in 11/24 studies, followed by hypothetical (9/24) and synthetic populations (3/24). The interventions consisted of a combination of strategies in most cases, including digital or manual contact tracing and non-pharmaceutical interventions (e.g. social distancing, lockdowns, mask wearing and hand hygiene). Recursive^19–22^ and bidirectional contact tracing^23^ resulted as more effective than forward-tracing alone, albeit leading to the quarantine of a substantial number of healthy individuals. In recursive contact tracing, not only direct contacts are traced but also contacts of contacts while in bidirectional contact tracing, reverse tracing identifies the parent case who infected a known case and the process continues to discover other cases related to the parent case. The interventions under study were compared with multiple hypothetical scenarios, comprising no intervention (no contact tracing, testing or social distancing), manual/DCT or combined interventions.

**Table 2:** Main characteristics of the modelling studies included in the systematic review on COVID-19 digital contact tracing

Refereces	Study setting	Type of model	Study population	Intervention	Comparisons	**Effectiveness of the intervention (*R*_0_** ^a^ **, *R*_eff_** ^b^ **)**
Abueg^28^	USA (Washington State)	Agent-based models	General population	Exposure notifications, non-pharmaceutical interventions	Multiple hypothetical scenarios with combinations of digital exposure notification, manual contact tracing, social distancing	*R*_eff_ not reported. An app adoption of 75% reduces the total number of infections by 56–73%, 73–79% and 67–81% and deaths by 52–70%, 69–78%, and 63–78% in three counties. Even at a low level of app adoption of 15%, there are meaningful reductions in infections, hospitalizations and deaths from COVID-19
Aleta^c^	USA	Agent-based models	Synthetic population of the Boston metropolitan area	(i) Unmitigated scenario (no interventions); (ii) LIFT scenario (the stay-at-home order is lifted after 8 weeks by reopening all work and community places, except for mass-gathering locations, a full listing of all the remaining restrictions 4 weeks later while schools will remain closed; (iii) LIFT and enhanced tracing scenario—LET (the stay-at-home order is lifted, symptomatic COVID-19 cases can be diagnosed and isolated at home and their household members are quarantined for 2 weeks)	Unmitigated scenario (LIFT) vs.^d^ two mitigation scenarios (LET)	LIFT scenario is able to temporarily abate the epidemic incidence, but does not prevent the resurgence of the epidemic and a second COVID-19 wave when the social-distancing measures are relaxed. Following the lifting of social distancing, the infection incidence starts to increase again, and the *R*_eff_, which was <1 with the intervention, increases up to 2.05 (95%CI: 1.73–2.47). In the LET scenario, when 40% or more of the contacts of the detected symptomatic infections are traced and they and their households are quarantined, the reduction in transmission effectively limits the possible resurgence of a second epidemic wave (*R*_eff_ after total reopening increases to 1.4)
Almagor^c^	UK	Agent-based models	Synthetic population derived from the 2011 UK Census	DCT,^e^ testing, self-isolation	Baseline scenario of ‘no tests and no CTA^f^^’^ vs.^d^ (i) testing without CTA^f^; (ii) CTA^f^ with a testing policy that prioritizes symptomatic cases; (iii) CTA^f^ with no priority to test symptomatic cases; (iv) CTA^f^ users’ low or high level of compliance with self-isolation	Irrespective of testing capacity, when symptomatic agents are prioritized for testing and the proportion of CTA^f^ users increases, overall infections decrease. The decrease is most substantial when testing is not limited. As larger fractions of society adopt the CTA,^f^ the spread of the virus is increasingly reduced, and therefore, the benefits extend to the wider population
Barrat^19^	France, Denmark	Compartmental model	Students, workers	Isolation, manual contact tracing, DCT,^e^ recursive contact tracing	The combined intervention of manual contact tracing and DCT^e^ vs.^d^ other interventions	Contact tracing is more efficient in a situation where the epidemic is already partially mitigated by other measures that keep *R*_0_ as low as possible, showing the importance of combining contact tracing with other measures such as masks or hand hygiene
Bicher^c^	Austria	Agent-based model	General population	Six different strategies: One strategy without tracing (no tracing); three strategies with location tracing (household tracing, workplace tracing, combined household and workplace tracing); two strategies of individual contact tracing (persons using a tracking device, e.g. smartphone)	Comparison of the six strategies	Isolation of household members is the most accurate measure and leads to the highest number of infections averted in relation to quarantined persons (quarantined per infection prevented, QpIp = 0.69). Temporary closing of only workplaces due to positive cases is the least accurate and the costliest of the modelled policies (QpIp = 4.15). Combining the two policies and adding leisure-time contact reduction also results in a costlier strategy, yet a greater reduction in infectivity can be reached because more secondary infections are found and isolated (QpIp = 2.22 for combined tracing, 3.2 for 50% individual tracing and 3.85 for 75% individual tracing)
Bradshaw^23^	nr	Stochastic branching-process model	Hypothetical population	DCT^e^ with/without manual tracing, isolation	Forward vs.^d^ bidirectional tracing: (i) manual tracing only; (ii) bidirectional manual tracing; (iii) bidirectional digital tracing; (iv) hybrid tracing: manual + digital tracing; (v) bidirectional hybrid tracing with 2-day manual window; (vi) bidirectional hybrid tracing with 6-day manual window; (vii) no tracing	Bidirectional tracing more than doubles the reduction in *R*_eff_ achieved by forward tracing alone. Expanding the manual tracing window to enable more effective bidirectional tracing or implementing high-uptake bidirectional digital exposure notification could substantially improve COVID-19 control. In the absence of bidirectional tracing, a hybrid approach offered few benefits over manual tracing, reducing *R*_eff_ by up to 0.06 in the low-uptake condition and 0.12 in the high-uptake condition compared to manual tracing alone. Digital exposure notification alone is unlikely to control the epidemic
Bulchandani^26^	nr	Stochastic branching-process model	Hypothetical population	DCT,^e^ quarantine of infected population	None	Digital immunity can be achieved (*R*_eff_ < 1) when mobile app uptake ranges 75–95%, regardless of the % of non-symptomatic transmission
Currie^30^	Australia	Dynamic aggregate-level model (modified SEIR^g^ model)	General population	Three testing scenarios: (i) maintaining testing at May 2020 levels until December 2020 (no tapering), (ii) testing levels tapering by 5% per month and (iii) testing levels tapering by 10% per month and two social distancing scenarios: (i) with a more rapid reduction; (ii) with a slower reduction maintained over time	Five scenarios with different levels of app uptake (0%, 27%, 40%, 61%, 80%): (i) baseline (decline in social distancing of 50% and a decline in testing intensity of 5% every month), (ii) slower easing of social distancing, (iii) rapid easing of social distancing, (iv) sustained diagnostic testing intensity, (v) reduced diagnostic testing intensity scenarios	The COVIDSafe app is a potentially valuable adjunct to testing and social distancing. If a high level of community uptake can be achieved, the app could have a mitigating effect on any new wave of COVID-19 by enhancing contact detection regardless of the presence or absence of individuals’ symptoms
Ferrari^c^	Italy	Compartmental model (SIR^h^)	General population from 110 Italian districts updated to 2016	DCT^e^	Different population densities across the 110 districts are compared	*R*_eff_ not considered. (i) Voluntary self-quarantine based on contact-tracing apps, with efficient case isolation, can give a relevant, contribution to epidemics mitigation/suppression; (ii) the success of this strategy can depend heavily on population density and transportation. Even if travel between districts is forbidden, the epidemics may still be significantly harder to contain in areas with very high population density (e.g. in Italy, the districts of Milano, Monza and Napoli)
Ferretti^29^	nr	General mathematical model	Hypothetical population	Isolation of symptomatic individuals, tracing and quarantining the contacts of symptomatic cases	Traditional manual contact tracing	Manual contact tracing is too slow, and personnel limitations prevent it from being scaled up when the epidemic grows beyond the early phase. The combination of isolation and contact tracing with mobile app/quarantining could bring *R*_0_ < 1 and therefore effectively control the epidemic. Immediate notification through a CTA^f^ could be sufficient to stop the epidemic if used by a sufficiently high proportion of the population. For a 3-day delay in notification assumed for manual contact tracing, no parameter combination leads to epidemic control
Firth^21^	UK	Epidemic network-based model	General population	DCT,^e^ quarantine	Primary and secondary contact tracing using GPS^i^ data	Isolation of individuals when they became symptomatic resulted in 66% (62–69%) of the population infected, and primary contact tracing resulted in 48% (42–54%) infected. Secondary contact tracing resulted in the smallest percentage (16%, 11–22%) of the population infected after 70 days. Thus, tracing and quarantining contacts of contacts was the most effective measure for controlling local COVID-19 outbreaks but required large numbers of individuals to be quarantined. This strategy is similar to introducing a local lockdown. Testing and releasing quarantined individuals reduced the numbers quarantined but also reduced the effectiveness of control measures. Combining contact tracing with other physical distancing measures could control the outbreak, while reducing the number of people in quarantine and the number of tests required
Hinch^22^	UK	Individual-based model	General population	DCT,^e^ physical distancing, generalized lockdowns	Six scenarios were compared: (i) no app; (ii) app without recursion, quarantine: index cases, their households, their contacts, release: everybody after 14 days from notification; (iii) app with recursion, quarantine: as scenario 2 + household members of contacts, release: as scenario 2; (iv) app with recursion and cluster release, quarantine: as scenario 3, release: as scenarios 2 and 3 + release of an index case cluster if nobody from the cluster develops symptoms within 5 days; (v) app with recursion and testing as follow-up, quarantine: as scenarios 3 and 4, release: as scenarios 2 and 3 + release of an index case cluster if index case had a negative test; (vi) app with recursion and notification upon testing, quarantine: contacts are notified only after index case tests positive, release: as scenarios 2 and 3	Under the assumptions of continued lockdown of people aged over 70, low smartphone use, high COVID-19 mortality, no use of the app in children aged under 10, the epidemic can be suppressed with 80% of all smartphone users using the app or 56% of the population overall
Kim^c^	nr	nr	Hypothetical population	DCT^e^	None	With 30% probability of transmission, about 40–60% of the population needs to be enrolled and 75–90% confirmed as infected individuals, for the measure to work in the absence of active social distancing measures
Kretzschmar^c^	Netherlands	Stochastic mathematical model	General population	(i) Contact tracing: conventional contact tracing and DCT^e^; (ii) physical distancing and isolation for symptomatic individuals	Strategies without contact tracing (physical distancing and isolation) vs.^d^ strategies with contact tracing (conventional and mobile app contact tracing with testing coverage between 20% and 100%)	In the most optimistic scenario (testing and tracing delays of 0 days and tracing coverage of 100%), with 40% of transmissions occurring before symptom onset, *R*_eff_ of 1.2 (with physical distancing only) will be reduced to 0.8 (95%CI 0.7–0.9) by adding contact tracing. A similar reduction can be achieved when testing and tracing coverage is reduced to 80% (*R*_eff_ 0.8, 95%CI 0.7–1.0). If the testing delay is 2 days, the tracing delay needs to be at most 1 day or tracing coverage needs to be at least 80% to keep *R*_eff_ < 1. If the testing delay becomes 3 days or longer, even perfect contact tracing (i.e. 100% testing and tracing coverage with no tracing delay) cannot bring *R*_eff_ < 1
Kucharski^c^	UK	Transmission model	General population	No control, contact tracing strategies (manual tracing, app-based tracing), testing, mass testing of all cases regardless of symptoms, self-isolation of symptomatic cases, quarantine	Baseline scenario (delay of symptom onset to isolation of 2.6 days, quarantine within 2 days for successfully manually traced contacts and immediately for app-based tracing, 90% of contacts assumed to adhere to quarantine) vs.^d^ various individual or combined scenarios	Strategies that combined isolation of symptomatic cases with tracing and quarantine of their contacts reduced *R*_eff_ more than mass testing or self-isolation alone. The effectiveness of these isolation and tracing strategies is further enhanced when combined with physical distancing measures, such as a reduction in work contacts, or a limit to the number of contacts made outside of home, school or work settings. The effectiveness of manual contact tracing strategies was highly dependent on how many contacts were successfully traced, with a high level of tracing required to ensure *R*_eff_ < 1. App-based tracing would also require a high level of coverage to ensure *R*_eff_ < 1 because both primary case and contacts would need to install and use the app
Moreno Lopez^24^	France	Agent-based model	Synthetic population (based on INSEE^j^ censuses)	DCT^e^ + testing and isolation of clinical cases and household members	Uncontrolled scenario (*R* = *R*_0_ = 3.1) vs.^d^ scenarios where the transmissibility is reduced because of the adoption of barrier measures (*R* reduced to 1.5)	The inclusion of DCT^e^ in all scenarios would increase the relative reduction to 35% with about 20% app adoption and to 66% with about 60% app adoption. Stronger reductions could be obtained with more efficient detection of clinical cases. Higher coverage among adults, playing a central role in COVID-19 transmission, yields an indirect benefit for the elderly
Nakamoto^25^	Japan	Compartmental model (SIR^h^)	General population	Scenarios (households, schools, workplaces, etc.) in which the epidemic is established and countermeasures such as contact tracing are employed to control the spread of COVID-19	Scenarios with different levels of app uptake	When the uptake rate for contact tracing app increases, the effective reproduction number decreases gradually. To meaningfully contain the spread of COVID-19 (i.e. *R*_eff_ < 1), approximately 90% participation of the population would be required
Nuzzo^34^	USA	Compartmental model (SEIR^g^)	Hypothetical population	DCT,^e^ targeted self-isolation	No intervention, universal shelter-in-place (entire population isolated for 50 days) vs.^d^ DCT^e^ with targeted self-isolation	No intervention leads to a high rate of SARS-CoV-2 infection. Both shelter-in-place and DCT^e^ achieve reductions in infected cases, but the first has the higher societal burden in terms of the number of individuals expected to be quarantined or isolated. A 50% adoption rate of digital contact tracing is comparable to a shelter-in-place order for 40% of the population and results in over 90% decrease in the peak number of infections
Peak^c^	nr	Stochastic branching-process model	General population	Individual quarantine or active monitoring of contacts (includes phone-based self-monitoring)	Short (mean 4.8 days; scenario 1) vs.^d^ long (mean 7.5 days; scenario 2) serial interval estimates	Manual contact tracing and automated monitoring under both serial interval scenarios, in a low feasibility setting: *R*_eff_ was rarely <1 under either individual quarantine or active monitoring. For short serial intervals, active monitoring resulted in only a 3% reduction in the *R*_eff_, whereas individual quarantine resulted in a 17% reduction. Tracing 10%, 50% or 90% of contacts in addition to physical distancing resulted in a median reduction in *R*_eff_ of 3.2%, 15% and 33%, respectively, for active monitoring, and 5.8%, 32% and 66%, respectively, for individual quarantine
Pollmann^20^	nr	Two types of deterministic models; two individual-based models with the MC^k^ simulation technique	Hypothetical population	DCT^e^, quarantine, testing, social distancing	COVID-19 outbreak was compared under different intervention protocols, focusing on DCT^e^ and DCT^e^ combined with random testing (administering a SARS-CoV-2 test to some fraction of the population at random, regardless of symptoms or contact history) and social distancing	An outbreak of COVID-19 cannot be fully controlled by DCT^e^ even if a large fraction of the population uses the DTC system. If interventions are started once an outbreak is already ongoing, DCT^e^ causes a large fraction of the healthy population to be traced and quarantined. DCT^e^ can be combined with other measures, such as face–mouth coverings, social distancing and/or random testing, to achieve outbreak control. The availability of fast testing, and coordination of test results with the DCT^e^ system, are crucial to allow symptomatic cases to become index cases for tracing and to release traced healthy contacts from quarantine. Recursive tracing is more efficient than one-step tracing (i.e. infectious contacts followed for one step)
Wallentin^c^	Austria	Agent-based model	General population (Salzburg)	Four scenarios: (i) continued lockdown; (ii) stepwise relaxation of the lockdown; (iii) relaxation of the lockdown paralleled with low, medium or high levels of DCT^e^; (iv) stepwise relaxation with monitoring and adaptive response	Comparison of the four scenarios	The success of contact tracing depends on how widely it is deployed. Low-level voluntary use of CTA^f^ shows no relevant effects on containing the virus. Contact tracing gets effective above 25% of contacts isolated within the first 3 days; then it allows for social and economic activity at high levels
Whaiduzzaman^31^	Australia	PPMF^l^	Hypothetical population	DCT^e^	Other mobile application frameworks (i.e. Tracetogheter, COVIDsafe)	In terms of privacy-preserving approaches (voluntary, data usage limitation, data destruction, minimal data collection and transparency), fog-based integrated solutions (risk check, infected/suspected data upload) and general design approaches (no geo-location trace, and minimal internet requirements), PPMF^l^ considers all these features. Fog-based integrated solutions are not present in any other digital framework
Wilmink^32^	USA	Compartmental model (SEIR^g^)	Hypothetical population	Real-time DCT^e^	DCT^e^ (wearable device) vs.^d^ conventional control methods (symptom-based monitoring, PCR^m^ testing, manual contact tracing)	DCT^e^ allows for rapid and effective identification and containment of potentially infected close contacts. The speed and efficiency of DCT^e^ led to 52% fewer cases than conventional methods. The manual contact tracing process is slow and has inherent time delays between confirming a case and finding a person’s contacts. These time delays give secondary contacts more time to transmit the virus even further in the facility. Manual contact tracing also relies on humans both for data collection and data entry, which increases the potential for inaccurate or incomplete results due to human error
Yasaka^27^	nr	Transmission graph (adapted SIR^h^ model)	Hypothetical population	DCT,^e^ quarantine	Other contact-tracing apps, especially those with location tracking	The app adoption rate is key to the impact on the extent of an outbreak. A peer-to-peer contact tracing app can be effective without 100% participation: even a 25% adoption would provide some suppression of the infection curve compared to no adoption. The use of location-based traffic detection algorithms may provide a more robust measure for estimating user real-time location at points of contact but presents many potential privacy concerns

a *R*_0_, basic reproduction number.

b *R*_eff_, effective reproduction number.

c Supplementary material S1.

d vs., versus.

e DCT, digital contact tracing.

f CTA, contact tracing app.

g SEIR, Susceptible-Exposed-Infectious-Recovered.

h SIR, Susceptible-Infectious-Recovered.

i GPS, Global Positioning System.

j INSEE, National Institute of Statistics and Economic Studies.

k MC, Monte Carlo; R, reproduction number.

l PPMF, integrated mobile-fog computing framework.

m PCR, polymerase chain reaction.

According to the studies, the adoption rate of contact tracing devices is the main factor impacting the spread of an outbreak. As larger proportions of the population adopt the devices, the spread of the virus is increasingly reduced; therefore, the benefits are extended to the wider population. To contain the spread of the coronavirus using only DCT, about 90% of the population is required to use the devices and strictly adhere to quarantining and testing protocols.^20^^,^^24–26^ However, DCT is still effective even with a 25% app adoption rate compared to no adoption. The use of geolocalization technologies, such as GPS, improves the estimation of the user’s real-time location but might present privacy issues.^27^ To achieve better outbreak control, DCT should be combined with other measures, such as social distancing, mask wearing or COVID-19 testing and traditional contact tracing. The availability of fast testing and timely coordination of test results with contact tracing are important for the effectiveness of the interventions. As recursive and bidirectional contact tracing often leads to a quarantine of a large proportion of the healthy population,^19–22^ among the benefits of combining DCT with other mitigation measures (e.g. traditional contact tracing) are higher and faster reduction of the epidemic size and lower societal and economic costs, in terms of quarantines and resources.^19–22^^,^^28^ Traditional contact tracing alone is not fast enough for the containment of the coronavirus. The delay between a case confirmation and contact identification is inevitable but could be mitigated by using a digital app. The lack of healthcare professionals is also a concern when the pandemic progresses.^29^

Regarding other aspects of the modelling studies, sensitivity analysis on uncertain parameters was performed in 13/24 studies. The communication technologies were essentially Bluetooth (12/24), GPS (2/24) and a combination of proximity-sensing applications (1/24); the technology was not specified in 7/24 studies. A clear definition of contact was reported in 15/24 studies and mostly included physical distancing, duration and frequency of the encounters. COVID-19 test specificity was included in the model in one study,^28^ while sensitivity was included in three studies.^23^^,^^28^^,^^30^ Privacy and security issues of the devices were also considered in the models (11/24). The data sources used for the model parameters were mainly literature studies, publicly available datasets from the national census and surveys; they were not reported in two studies.^31^^,^^32^ The codes used for the analysis are publicly available for 15/24 studies, mostly published on GitHub.

### Quality assessment

The majority (9/13) of the population-based studies achieved a moderate quality level (with one weak section rating). Two studies obtained a strong global rating for not achieving weak ratings in any section. Weak global ratings were obtained by two studies due to bias in the study design and blinding sections. The sections ‘confounders’ and ‘withdrawals and dropouts’ were not applicable in all studies.

All modelling studies (table 3), except four, had a structured abstract summarizing all important elements and the introduction sections provided a broader context and relevance of the study. The target populations and subgroups were not always well analyzed or specified (8/24), as well as the setting (6/24). The analytic methods supporting the model were not described in one study.^27^ Values, ranges, references and probability distributions for all parameters were not reported in three of the articles and the sources of uncertainty were also lacking (4/24). The funding source was the least reported item (11/24), the role of the funding body was also missing in 6/24 articles. Three studies did not provide a conflict of interest statement.

**Table 3:** Quality assessment of modelling studies on COVID-19 digital contact tracing performed with the CHEERS^a^ checklist

References	2. Structured abstract	3. Introduction (context, relevance)	4. Target population, subgroups	5. Setting	7. Comparators	15. Choice of model	16. Assumptions	17. Analytical methods	18a. Values, ranges, references for parameters	18b. Sources for uncertainty	22a. Key findings	22b. Limitations, generalizability	23. Funding, role of funders	24. Conflict of interest
Abueg^28^	✓	✓	✓	✓	✓	✓	✓	✓	✓	Partial (no input values)	✓	✓	No	✓
Aleta^b^	✓	✓	✓	✓	✓	✓	✓	✓	✓	✓	✓	✓	✓	✓
Almagor^b^	Partial (not detailed)	✓	✓	Partial (no details)	✓	✓	✓	✓	✓	✓	✓	✓	✓	✓
Barrat^19^	✓	✓	✓	Partial	✓	✓	✓	✓	Partial (no ranges)	✓	✓	✓	Partial (no role of funder)	✓
Bicher^b^	✓	✓	✓	✓	✓	✓	✓	✓	✓	✓	✓	✓	✓	✓
Bradshaw^23^	Partial (not detailed)	✓	No	No	✓	✓	✓	✓	✓	✓	✓	✓	No	✓
Bulchandani^26^	Partial (not detailed)	✓	No (no details)	No (no setting)	No	✓	✓	Partial (uncertainty, heterogeneity not handled)	Partial (no complete list of parameters and sources)	Partial (no details)	✓	Partial (no methodological limitations)	No	No
Currie^30^	✓	✓	✓	✓	✓	✓	✓	Partial (no sensitivity analysis)	✓	No (no range for uncertainty values)		✓	No	✓
Ferrari^b^	✓	✓	✓	✓	✓	✓	✓	✓	✓	✓	✓	✓	Partial (no role of funder)	✓
Ferretti^29^	✓	✓	No	Partial (no setting, data calibration for China)	✓	✓	✓	✓	✓	✓	✓	✓	✓	✓
Firth^21^	✓	✓	✓	✓	✓	✓	✓	✓	✓	✓	✓	✓	Partial (no role of funder)	✓
Hinch^22^	No	✓	✓	✓	✓	✓	✓	✓	✓	✓	✓	✓	No	No
Kim^b^	✓	✓	No	No (no setting)	No	✓	✓	Partial (uncertainty, heterogeneity not handled)	Partial (no complete list of parameters and sources)	Partial (no details)	✓	✓	Partial (no role of funder)	✓
Kretzschmar^b^	✓	✓	Partial (no details)	✓	✓	✓	✓	✓	Partial (no complete list of parameters)	✓	✓	✓	✓	✓
Kucharski^b^	✓	✓	✓	✓	✓	✓	✓	✓	✓	✓	✓	✓	Partial (no role of funder)	✓
Moreno Lopez^24^	✓	✓	✓	✓	✓	✓	✓	✓	✓	✓	✓	✓	Partial (no role of funder)	✓
Nakamoto^25^	✓	✓	✓	✓	✓	✓	✓	Partial (uncertainty, heterogeneity not handled)	No	No	✓	✓	No	✓
Nuzzo^34^	✓	✓	Partial (no details)	✓	✓	✓	✓	Partial (uncertainty, heterogeneity not handled)	Partial (no complete list of parameter values)	No	✓	✓	No	✓
Peak, 2020^b^	✓	✓	No	No	✓	Partial (no figure of model structure)	✓	✓	✓	✓	✓	✓	✓	✓
Pollmann^20^	✓	✓	No	No	✓	✓	✓	✓	✓	✓	✓	✓	✓	✓
Wallentin^b^	Partial (no setting)	✓	✓	✓	✓	✓	✓	✓	✓	✓	✓	Partial (all limitations not reported)	No	✓
Whaiduzzaman^31^	✓	✓	No	No	✓	✓	✓	Partial (uncertainty, heterogeneity not handled)	No	No	✓	Partial (no limitations)	No	No
Wilmink^32^	✓	✓	✓	✓	✓	✓	✓	Partial (no sensitivity analysis)	Partial (no main values, ranges)	No	✓	✓	No	✓
Yasaka^27^	✓	✓	No	No	✓	✓	Partial (assumptions not considered)	No	No	No	✓	✓	No	✓

a CHEERS, Consolidated Health Economic Evaluation Reporting Standards.

b Supplementary material S1.

### Limitations

Limitations of the systematic review are inherent to the availability of some records only as preprints and to the lack of full-text articles that were eliminated from the study. Although this could impact the findings, the included records were informative and of medium to high quality, allowing a detailed assessment of the studies. Out of the two excluded preprints during the full-text assessment, only Plank et al. has been published in its definitive form in 2022. This raises concerns regarding the preprint’s overall quality and its potential impact on the systematic reviews which encompassed it.^7^^,^^8^ Given that our review focused on the initial 18 months of the pandemic, the study published in 2022 was excluded. Nevertheless, its findings align with our research in terms of the effectiveness of DCT when combined with other strategies and the advantages of a high adoption rate of digital tools.

The uptake rates of the tools in the population-based studies (i.e. percentage of downloads, active app users and positive test among app users) were not referred to the entire population of the country under consideration but to the sample size of the study, which was not specified in all records. Only two studies reported data related to the entire population: the Swiss population^2^ and the residents of England and Wales.^33^ Therefore, the percentages of downloads were reported or calculable in five studies,^2^^,^^14–16^^,^^33^ and in six studies each for active users^2–4^^,^^14^^,^^15^^,^^33^ and positive test among active users.^2^^,^^3^^,^^14^^,^^16^^,^^17^^,^^33^ The information was also reported differently in each study (e.g. the proportion of enrollees that accepted to download and use the app, percentage change of app users during the study and number or percentage of notified cases). This rendered synthesis and comparison of the uptake rate difficult. However, the effectiveness of contact tracing and the level of implementation of the devices in specific settings were assessed with other available indicators, such as the number of identified close contacts of COVID‐19 cases, close contacts per index case, laboratory-confirmed COVID-19 cases detected from close contacts, the reproduction number and reduction of COVID-19 infections. A meta-analysis was not performed due to the heterogeneity of the studies.

## Discussion

This study evaluated the role of digital tools deployed for contact tracing during the recent public health emergency, taking into consideration their effectiveness and impact on the population and healthcare providers. DCT is considered a valuable approach to limit the spread of SARS-CoV-2 but should be combined with other preventive measures and the population uptake must be high to contain the outbreaks. Recursive and bidirectional contact tracing resulted as more effective than forward-tracing alone.^19–22^ The combination of digital and traditional contact tracing renders the tracing process faster, reduces the workload for healthcare professionals and mitigates the societal and economic cost due to the quarantine of healthy subjects and required resources.^19^^,^^20^^,^^28^^,^^29^ DCT alone could effectively contain the pandemic only if the entire population uses digital devices and strictly adheres to preventive measures (e.g. social distancing, quarantine, hand washing, mask covering, testing and vaccination).^20^^,^^24–26^ However, even with a lower uptake rate, DCT still reduces the epidemic size compared to no adoption of the apps.^27^ It should be noted that two indicators used to evaluate the effectiveness of DCT (i.e. the number of close contacts of COVID-19 cases/close contacts per index case; laboratory-confirmed COVID-19 cases detected from close contacts) may be affected by risk mitigation measures (e.g. quarantine, social distancing, testing, vaccination), which were adopted according to the changing epidemic situation and may have in part affected the results of the included studies. The range of the cases from close contacts reported by the studies is often very large. This could also be due to the pandemic period, thus to more or less aggressive circulating virus variants and the related risk mitigation measures adopted by the countries.

The main barriers to the wide implementation of emerging devices are the adoption rate^2^^,^^24^^,^^27^^,^^28^^,^^33^^,^^34^ and privacy and security issues.^2^^,^^13^^,^^15^^,^^16^^,^^27^^,^^31^^,^^33^ Several data security and privacy breaches have been registered worldwide,^35^ and were related to data storage on a central server in centralized protocols presenting security and other technical limitations. Contrarily, decentralized systems allow data to be stored on individual devices avoiding such risk. Although some EU Member States modified the first versions of the applications, switched to decentralized protocols (e.g. Austria, Italy, Ireland, Germany, the UK), and nominated national data controllers according to the General Data Protection Regulation (GDPR) and recommendations of the EU Commission, the uptake rate of the new devices remained low/medium across Europe.^6^^,^^35^ Other barriers have been identified in the literature, such as institutional distrust, low health literacy and lack of expertise of healthcare personnel in information technology.^6^ Institutional distrust has worsened during the recent pandemic^36^ and could be caused by the mandatory use of monitoring apps (e.g. South Korea, Singapore, Taiwan)^13^^,^^16^ or mandatory COVID-19 vaccination in some countries (e.g. Austria, Greece, Italy, Indonesia, Malaysia, Ecuador, Costa Rica), but also by infodemics (both misinformation and disinformation) related to COVID-19.^6^^,^^35^^,^^37^ The implementation of digital devices according to the GDPR and public health interventions to improve health literacy and fight disinformation, as well as capacity building courses in information technologies addressing healthcare providers, could enhance the adoption rate of the new digital solutions and improve countries’ emergency preparedness.

The lack of transparency in code sharing is another barrier to the uptake rate of the new devices.^6^^,^^38^ The majority of the modelling studies included in the systematic review have published the code used for the analysis, facilitating the comparison of source codes across countries.

The characteristics of DCT have been assessed in other literature reviews; in particular, effectiveness,^7^^,^^8^^,^^39^ ethical considerations^40^ and security and privacy issues,^8^ confirming the findings of the present study. However, systematic reviews summarizing the findings about data privacy and security, community uptake and the overall effectiveness of DCT in one study were lacking.^7^ The present study considers all these, and in addition, includes several population-based and modelling studies comparing traditional and DCT that were not sufficiently represented or evaluated in previous reviews.^8^ Two recent reviews by Juneau et al.^39^ and Pozo-Martin et al. (2023) (Supplementary material S1) have explored the effectiveness of contact tracing in modelling and observational studies. Nevertheless, they did not delve into ethical, security and privacy issues.

In conclusion, the included studies provided evidence of the effectiveness of DCT, especially when combined with other preventive measures. The combination of digital and traditional contact tracing has a positive impact on societal and economic costs. The uptake rate, as well as privacy and security issues, are the main barriers to the wide adoption of the new tools, and thus for the containment of the pandemic. The integration of digital devices into routine epidemiological investigations also depends on the availability of healthcare professionals, especially in the latter stages of the pandemic. Emergency preparedness could be enhanced by public health digitalization, which should be among the top priorities in all countries.

## Supplementary Material

ckae039_Supplementary_Data

## Data Availability

The data underlying this article are available in the article and in its online supplementary material. Key points DCT, in combination with other containment measures (e.g. mask usage, social distancing), is effective in curbing the COVID-19 pandemic Uptake rate, security and privacy issues have limited the use of DCT devices and need to be addressed to enhance the integration of digital devices into conventional epidemiological investigations Healthcare digitalization should be a priority on countries’ political agenda to improve epidemic response preparedness Health information systems should include linkage with geolocalization data in order to detect an individual (by definition healthy) in place and time
