# Supplementary material for: The role of digital tools and emerging devices in COVID-19 contact tracing during the first 18 months of the pandemic: a systematic review
Source: Eur J Public Health. 2024 Jul 1;34(Suppl 1):i11–28. doi: 10.1093/eurpub/ckae039 (PMC11215323; doi:10.1093/eurpub/ckae039)
Supplement: ckae039_Supplementary_Data [file ckae039_supplementary_data.zip › ejph-2023-06-phis-0277-File007.pdf]

Supplementary material 2. Definition of contact tracing and related terms used in the systematic review on the role of digital tools and emerging devices in Covid-19 contact tracing during the first 18 months of the pandemic

**Contact tracing**	the process of rapid identification, assessment and management of individuals who have recently been in contact with someone diagnosed with an infectious disease, in order to prevent additional transmission[1]
**Traditional (forward or one-step) contact tracing**	aims to identify individuals that were infected by recent contact with someone diagnosed with an infectious disease (tracing to whom disease spreads)[1]
**Manual contact tracing**	involves public health staff that perform a detailed epidemiological investigation to identify the contacts of an infected individual, interview them (in person or by telephone) to get additional context about the exposure, and monitor their symptoms[2]
**Digital contact tracing**	contact tracing supported by digital proximity tracing or mobile applications (apps) that can detect the duration and distance of contact between users of the app. In case of a positive test, the app notifies users who have been in close proximity to the infected individual[1]. Other functions of the apps are Covid-19 symptoms monitoring, general health functionalities, and booking for Covid-19 testing[3]
**Recursive (two-step or secondary) contact tracing**	contact tracing of individuals situated two hops away from a confirmed case, along the digitally sensed proximity network (i.e., tracing not only direct contacts but also contacts of contacts and so on)[4,5]
**Bidirectional contact tracing**	uses backward contact tracing to identify the source of infection (the parent case or infector) who infected a known case, then continues tracing to iteratively discover other cases related to the infector[6]

References

1. European Centre for Disease Prevention and Control. Contact tracing in the European Union: public health management of persons, including healthcare workers, who have had contact with COVID-19 cases – fourth update, 28 October 2021. Stockholm: ECDC; 2021
2. World Health Organization. Contact tracing in the context of COVID-19: Interim guidance, May 2020. https://apps.who.int/iris/bitstream/handle/10665/332049/WHO-2019-nCoV-Contact_Tracing-2020.1-eng.pdf
3. Unim B, Schutte N, Thissen M, Palmieri L. Innovative Methods Used in Monitoring COVID-19 in Europe: A Multinational Study. Int J Environ Res Public Health. 2022;20(1):564. doi: 10.3390/ijerph20010564.

4. Klinkenberg D, Fraser C, Heesterbeek H. The effectiveness of contact tracing in emerging epidemics. PLoS One. 2006;1(1):e12. doi: 10.1371/journal.pone.0000012
5. Barrat A, Cattuto C, Kivelä M, Lehmann S, Saramäki J. Effect of manual and digital contact tracing on COVID-19 outbreaks: a study on empirical contact data. J R Soc Interface. 2021;18(178):20201000. doi: 10.1098/rsif.2020.1000
6. Bradshaw WJ, Alley EC, Huggins JH, Lloyd AL, Esvelt KM. Bidirectional contact tracing could dramatically improve COVID-19 control. Nat Commun. 2021;12(1):232. doi: 10.1038/s41467-020-20325-7
