# Supplementary material for: The role of digital tools and emerging devices in COVID-19 contact tracing during the first 18 months of the pandemic: a systematic review
Source: Eur J Public Health. 2024 Jul 1;34(Suppl 1):i11–28. doi: 10.1093/eurpub/ckae039 (PMC11215323; doi:10.1093/eurpub/ckae039)
Supplement: ckae039_Supplementary_Data [file ckae039_supplementary_data.zip › ejph-2023-06-phis-0277-File006.pdf]

Supplementary material 1: extra references

- Barrett PM, Bambury N, Kelly L, Condon R, Crompton J, Sheahan A, on behalf of the regional Department of Public Health. Measuring the effectiveness of an automated text messaging active surveillance system for COVID-19 in the south of Ireland, March to April 2020. Euro Surveill. 2020;25(23):pii=2000972. https://doi.org/10.2807/1560-7917.ES.2020.25.23.2000972
- Fetzer T, Graeber T. Measuring the scientific effectiveness of contact tracing: Evidence from a natural experiment. Proc Natl Acad Sci U S A. 2021; 118(33):e2100814118. doi: 10.1073/pnas.2100814118
- Kwon KS, Park JI, Park YJ, Jung DM, Ryu KW, Lee JH. Evidence of Long-Distance Droplet Transmission of SARS-CoV-2 by Direct Air Flow in a Restaurant in Korea. J Korean Med Sci. 2020;35(46):e415. doi: 10.3346/jkms.2020.35.e415. Erratum in: J Korean Med Sci. 2021;36(2):e23
- Aleta A, Martín-Corral D, Pastore Y, et al. Modelling the impact of testing, contact tracing and household quarantine on second waves of COVID-19. Nat Hum Behav. 2020;4(9):964-971. doi: 10.1038/s41562-020-0931-9.
- Almagor J, Picascia S. Exploring the effectiveness of a COVID-19 contact tracing app using an agent-based model. Sci Rep. 2020;10(1):22235. doi: 10.1038/s41598-020-79000-y.
- Bicher M, Rippinger C, Urach C, Brunmeir D, Siebert U, Popper N. Evaluation of Contact-Tracing Policies against the Spread of SARS-CoV-2 in Austria: An Agent-Based Simulation. Med Decis Making. 2021; 41(8):1017-1032. doi: 10.1177/0272989X211013306
- Ferrari A, Santus E, Cirillo D, et al. Simulating SARS-CoV-2 epidemics by region-specific variables and modeling contact tracing app containment. NPJ Digit Med. 2021;4(1):9. doi: 10.1038/s41746-020-00374-4.

- Kim H, Paul A. Automated contact tracing: a game of big numbers in the time of COVID-19. J R Soc Interface. 2021; 18(175):20200954. doi: 10.1098/rsif.2020.0954
- Kretzschmar ME, Rozhnova G, Bootsma MCJ, et al. Impact of delays on effectiveness of contact tracing strategies for COVID-19: a modelling study. Lancet Public Health 2020; 5(8): e452–e459. http://dx.doi.org/10.1016/S2468-2667(20)30157-2.
- Peak CM, Kahn R, Grad YH, et al. Individual quarantine versus active monitoring of contacts for the mitigation of COVID-19: a modelling study. Lancet Infect Dis. 2020; 20(9):1025-1033. doi: 10.1016/S1473-3099(20)30361-3.
- Wallentin G, Kaziyeva D, Reibersdorfer-Adelsberger E. COVID-19 Intervention Scenarios for a Long-term Disease Management. Int J Health Policy Manag. 2020; 9(12):508-516. doi: 10.34172/ijhpm.2020.130.
- Kucharski AJ, Klepac P, Conlan AJK, et al; CMMID COVID-19 working group. Effectiveness of isolation, testing, contact tracing, and physical distancing on reducing transmission of SARS-CoV-2 in different settings: a mathematical modelling study. Lancet Infect Dis. 2020; 20(10):1151-1160. doi: 10.1016/S1473-3099(20)30457-6
- Garg L, Chukwu E, Nasser N, Chakraborty C, Garg G. Anonymity Preserving IoT-Based COVID-19 and Other Infectious Disease Contact Tracing Model. IEEE Access. 2020; 8:159402-159414. doi: 10.1109/ACCESS.2020.3020513
- Altshuler T, Hershkovitz R. Digital contact tracing and the coronavirus: Israeli and comparative perspectives.The Brookings Institution, 2020. https://www.brookings.edu/wp-content/uploads/2020/08/FP_20200803_digital_contact_tracing.pdf
- World Health Organization. Contact tracing and quarantine in the context of the Omicron SARS-CoV-2 variant: Interim guidance. Geneva, 2022. https://www.ncbi.nlm.nih.gov/books/NBK591483/pdf/Bookshelf_NBK591483.pdf

- Pozo-Martin F, Beltran Sanchez MA, Müller SA, Diaconu V, Weil K, El Bcheraoui C. Comparative effectiveness of contact tracing interventions in the context of the COVID-19 pandemic: a systematic review. Eur J Epidemiol. 2023; 38(3):243-266. doi: 10.1007/s10654-023-00963-z.
- Plank MJ, James A, Lustig A, Steyn N, Binny RN, Hendy SC. Potential reduction in transmission of COVID-19 by digital contact tracing systems: a modelling study. Math Med Biol. 2022; 39(2):156-168. doi: 10.1093/imammb/dqac002. PMID: 35290447.
